# Telehealth memory clinics in primary healthcare: real-world experiences from low-resource settings in Greece

**DOI:** 10.3389/frdem.2024.1477242

**Published:** 2024-11-27

**Authors:** Eleutheria Aggeletaki, Vasileios Stamos, Eleni Konidari, Apostolos Efkarpidis, Anna Petrou, Kalliopi Savvopoulou, Evangelia Kontogianni, Konstantinos Tsimpanis, Theofanis Vorvolakos, Antonios Politis, Panagiotis Alexopoulos

**Affiliations:** ^1^Nursing Services Department, General Hospital of Syros "Vardakeio and Proio", Syros, Greece; ^2^Primary Healthcare Center of Erymanthia, Erymanthia, Greece; ^3^Mental Health Services, Patras University Hospital, Faculty of Medicine, School of Health Sciences, University of Patras, Patras, Greece; ^4^1st Department of Psychiatry, Eginition Hospital, National and Kapodistrian University of Athens, Athens, Greece; ^5^Department of Informatics and Telecommunications, National and Kapodistrian University of Athens, Athens, Greece; ^6^Department of Psychiatry, University General Hospital of Alexandroupolis, School of Health Sciences, Democritus University of Thrace, Alexandroupolis, Greece; ^7^Division of Geriatric Psychiatry and Neuropsychiatry, Department of Psychiatry, Johns Hopkins Medical School, Baltimore, MD, United States; ^8^Global Brain Health Institute, School of Medicine, Trinity College Dublin, The University of Dublin, Dublin, Ireland; ^9^Department of Psychiatry and Psychotherapy, Klinikum rechts der Isar, Technical University of Munich, Munich, Germany; ^10^Patras Dementia Day Care Centre, Patras, Greece

**Keywords:** memory clinics, low-resource settings, low- and middle-income countries, telemedicine, dementia, mild cognitive impairment

## Abstract

**Background:**

The role of primary healthcare is pivotal in the management of the surge of dementia prevalence particularly in low-resource areas. In this study, two telehealth-based memory clinics in primary healthcare operating within the frames of the INTegRated InterveNtion of pSychogerIatric Care (INTRINSIC) are presented.

**Methods:**

The first clinic, which is led by a general practitioner, operates at a primary healthcare center in a semi-mountainous area and closely collaborates with the geriatric psychiatry outpatient clinic of the Patras University General Hospital via a telehealth medicine platform. The second clinic is embedded at the General Hospital Center for Interconnected Psychiatric Support on the island of Syros, is led by registered nurses, and is interconnected with the geriatric psychiatry unit at the Eginition University Hospital in Athens.

**Results:**

Both memory clinics are in their infancy. At the general practitioner-led memory clinic, 13 beneficiaries were assessed and treated during the first 6 months of its operation. Cognitive decline and depressive and/or anxiety symptoms were detected in 10 and eight individuals, respectively. In 9 of the 27 beneficiaries of the registered nurse-led memory clinic, either mild cognitive impairment or dementia was diagnosed, while affective and/or anxiety symptoms were detected in almost all of them. Of note, only 14 beneficiaries of both clinics had received a diagnosis of a mental or neurocognitive disorder prior to their assessment at the memory clinics.

**Conclusion:**

Developing memory clinics in primary healthcare may be a pragmatic strategy to improve access of older adults living in low-resource areas to cognitive healthcare services.

## Introduction

As the global population ages, the incidence of neurocognitive disorders, including dementia, is increasing (GBD 2019 Dementia Forecasting Collaborators, 2022). According to the World Health Organization, by 2030, one in six people will be over 60 years old, resulting in more than 1.4 billion older adults worldwide (World Health Organization, 2017). Dementia, affecting over 55 million people worldwide, is the seventh leading cause of death (Shin, 2022), while Alzheimer’s disease, the most common cause of dementia, results in 36.3 million years lived with disability and is among the 25 leading causes of years lived with disability globally (Ferrari et al., 2023). The majority of people affected by dementia reside in middle- and low-income countries, and over 60% of those with dementia reside in regions with limited access to healthcare and social services, posing significant challenges to healthcare systems (Prince et al., 2015; Panagiotopoulos and Kaliampakos, 2019). By 2030, an estimated 40 million additional healthcare and social workers will be needed globally to manage dementia (UNDP, 2017).

The role of primary healthcare is pivotal in managing the surge of dementia cases across the globe. Dementia healthcare services are predominantly available in secondary and tertiary care settings in metropolitan areas, leaving older adults in low-resource areas, including islands, without access to essential mental and cognitive healthcare (Lahana et al., 2011; Arsenault-Lapierre et al., 2023; Kalaria et al., 2024). Of note, remoteness and insularity are negatively correlated with the availability of healthcare for older adults and educational services (Panagiotopoulos and Kaliampakos, 2019). This lack of healthcare access places additional strain on secondary and tertiary healthcare facilities (Alexopoulos et al., 2020). Primary healthcare has the potential to play a fundamental role in the timely and accurate diagnosis of dementia and in providing post-diagnostic care (Thompson et al., 2023). Training and supporting primary healthcare professionals in dementia care are crucial for meeting the needs of older individuals with neurocognitive disorders residing in low-resource settings (Krause et al., 2022). Telehealth may facilitate the integration of memory clinic services into primary healthcare in such settings (Sorinmade et al., 2020; AbdulRahman et al., 2022).

The INTegRated InterveNtion of pSychogerIatric Care (INTRINSIC) services are telehealth-based mental and cognitive health services for older adults provided at primary healthcare centers in low-resource settings in Greece. They were founded in 2022 according to a governmental plan for improving mental healthcare for people living in remote areas (Politis et al., 2023). Consisting of a network of collaborating primary healthcare centers and geriatric psychiatry units of university hospitals, the INTRINSIC services facilitate early detection, monitoring, and management of age-related brain diseases among older adults in such areas. Multiprofessional, psychogeriatric teams formed at primary healthcare centers in remote areas consisted of three to four healthcare professionals with a special interest and skills in geriatric mental health. Utilizing a digital platform, INTRINSIC offers training, support, and service development for medical and non-medical primary healthcare professionals. The INTRINSIC services are structured around six key pillars (Politis et al., 2023), i.e., (i) a digital platform, (ii) a comprehensive surveillance system for cognitive, behavioral, and mental health risk factors in older adults, (iii) auditory and vision assessments, performed by primary healthcare professionals trained by sensory therapists, (iv) pharmacological and psychosocial support, (v) a pragmatic psychotherapeutic intervention based on modified problem adaptation therapy (M-PATH; Kanellopoulos et al., 2020), and (vi) community involvement in designing and adjusting the services to local community needs.

Building on the INTRINSIC services and locally available resources and following a bottom-up approach, two pilot models of primary healthcare-based memory clinics have been developed in low-resource settings, i.e., a general practitioner (GP)-led memory clinic (GPMemo) and a registered nurse-led one (RNMemo; World Health Organization and Wonca Working Party on Mental Health, 2008). This brief research report aims to provide an overview of the diagnostic and therapeutic procedures of each clinic and describe the clinical characteristics of their beneficiaries in the first 6 months of the operation of the two clinics.

## Methods

### General practitioner-led memory clinic model (GPMemo)

The GPMemo is implemented at the Primary Healthcare Center of Erymanthia, located in a semi-mountainous area in Western Greece, which serves a population of 2,896 people aged 60 or older. The clinic is coordinated by a GP who has completed the INTRINSIC online educational program for providing essential mental and cognitive health services for older adults in primary healthcare settings. This memory clinic collaborates closely with the geriatric psychiatry unit of the Patras University General Hospital.

The beneficiaries of the GPMemo are individuals aged 60 and over who are referred to it due to cognitive complaints by primary healthcare professionals in the area. The diagnostic workup aims at clarifying if cognitive impairment is present, detecting its cause, initiating the necessary therapeutic interventions, and providing post-diagnostic care and follow-up assessments, so that there is rarely a need for referral to the memory clinic of the regional university hospital. Special attention is paid to the management of risk factors of cognitive decline such as sleep disturbances, diabetes mellitus, hypertension, dyslipidemia, cardiovascular disease, falls, and hearing difficulties. Additionally, emphasis is placed on reducing and ideally ceasing the use of benzodiazepine, alcohol consumption, and smoking (Livingston et al., 2020).

The workup takes place within two visits to the memory clinic. During the first visit, the GP collects a complete medical, neurological, psychiatric, and pharmaceutical history, including dosage and start date of medicines, a history of any prior mental and/or neurological disorders, and demographic data (age, education, marital status, occupation, etc.). A full clinical examination is performed, including vital signs, ECG, and a neurological assessment for signs of parkinsonism, coordination difficulties, motor issues, and eye movement control. Available, recent blood test results are reviewed, including thyroid hormones, folic acid, vitamin B12, liver enzymes (SGOT and SGPT), renal function (Cr, urea, and eGFR), and electrolytes. If no recent laboratory test results are available or if tests need to be repeated, they are prescribed by the GP. If recent brain imaging (CT or MRI) is not available, it is prescribed and special attention is paid to the presence of white matter lesions (Fazekas score) and middle temporal lobe atrophy (Scheltens score; Wahlund, 2016). The flexible neuropsychiatric assessment includes the Mini-Cog (Fage et al., 2021), the 2-item Generalized Anxiety Disorder (GAD-2) questionnaire (Sapra et al., 2020), and the Patient Health Questionnaire-2 (PHQ-2; Suhr and Angers, 2019) from the INTRINSIC diagnostic program. It also incorporates the Mini-Mental State Examination (MMSE; Kourtesis et al., 2020) and/or the Montreal Cognitive Assessment (MoCA; Poptsi et al., 2019; Dautzenberg et al., 2021), the Frontal Assessment Battery (FAB; Slachevsky et al., 2004), the Word Finding Disorder Test (WoFi; Georgiou et al., 2023), and the 15-item Geriatric Depression Scale-15 (GDS-15; Fountoulakis et al., 1999). The GP completes the Neuropsychiatric Inventory (NPI; Politis et al., 2004) and the Bristol Activities of Daily Living Scale (BADLS), using information from the care partner of the beneficiary. After the first visit, the GP consults the geriatric psychiatrist via HERMES videoconferencing to establish a clinical diagnosis according to the DSM-5 diagnostic criteria (Sachdev et al., 2014) and develop an appropriate management and treatment plan. If extensive neuropsychological or specific laboratory or imaging evaluation is necessary, or hospitalization is inevitable for instance due to exacerbation of neuropsychiatric symptoms, the beneficiary is referred to the relevant department of the Patras University Hospital by a psychiatrist of the geriatric psychiatry unit interconnected with the GPMemo, so that diagnostic and care continuity is safeguarded.

The second visit encompasses the discussion of the results of the neuropsychiatric assessment under consideration of the clinical examination, brain imaging, and blood test findings and the development of a treatment plan in line with the principles of evidence-based medicine and beneficiary’s and care partner’s values (Alexopoulos et al., 2024). Treatment strategies include pharmacological interventions, such as treatment with cholinesterase inhibitors, memantine, antidepressants, melatonin, and low-dosed atypical antipsychotics, if other strategies have proven unsuccessful, as well as the management of conditions such as diabetes, dyslipidemia, and hypertension. Individualized non-pharmacological recommendations mainly pertain to an increase in physical activity, cognitive stimulation, and social empowerment. In addition, personalized counseling is provided to care partners and family members of people with neurocognitive disorders. Beneficiaries receive a detailed medical note with the findings of the neuropsychiatric assessment, the established diagnosis, and the treatment plan.

### Registered nurse-led memory clinic model (RNMemo)

The Center for Interconnected Psychiatric Support (CIPSY) is based at the General Hospital of Syros, part of the Cyclades island group in the Aegean Sea. In Syros, there are 6,642 residents aged 60 years or older. The CIPSY memory clinic is run by three registered nurses (RNMemo) and focuses on the assessment, treatment, and monitoring of older adults’ cognitive health. CIPSY collaborates with the geriatric psychiatry unit of the 1st Department of Psychiatry of the National and Kapodistrian University of Athens, which is based at the Eginition University Hospital.

Individuals expressing concerns regarding their cognitive health are informed about the RNMemo and are referred to the INTRINSIC services. Demographic data (age, occupation, education, and marital status), as well as data related to medical history, including prior mental and/or neurological disorders and current medication, are collected. The cognitive assessment relies on the Mini-Cog (Fage et al., 2021) and the Montreal Cognitive Assessment (MoCA), while the presence of depressive and anxiety symptoms is determined with the PHQ-2, the GDS-15, and the Beck Anxiety Inventory (BAI). If necessary, additional information is obtained from care partners using the Neuropsychiatric Inventory (NPI).

At the end of the diagnostic workup, RNMemo healthcare professionals discuss the findings with a geriatric psychiatrist via HERMES videoconferencing to determine whether imaging/laboratory tests are required, to establish the clinical diagnosis according to the DSM-5 diagnostic criteria and develop an appropriate treatment plan, if necessary. The geriatric psychiatrist discusses the findings and the recommended treatment plan with the beneficiary and their care partner via teleconferencing through the digital platform of the INTRINSIC services, ensuring that the adherence of the beneficiary to the plan is safeguarded. In case further assessments or admission to the hospital are necessary, the service user is referred to the relevant sections of the General Hospital of Syros and/or health services of the University of Athens by a psychiatrist of the geriatric psychiatry unit of the Eginition Hospital. In addition to pharmacotherapy, at the RNMemo, the following individual or group non-pharmacological interventions for both people with neurocognitive disorders and their care partners are offered.

- The Memory Empowerment Groups aim at enhancing cognitive functions through activities such as speech exercises, card games, and social interaction.
- Problem Adaptation Therapy (PATH) focuses on the patients’ ecosystem (i.e., the beneficiary, the care partner, and the home environment). The PATH aims at reducing depressive symptoms in older adults with or without minor neurocognitive disorders and their care partners by shifting the balance of positive–negative emotions in favor of the former ones and facilitating problem-solving and adaptive functioning. Each PATH session lasts approximately 50 min and is conducted by trained nurses, and monthly supervised by PATH experts.
- Care partner training includes seminars to educate and support care partners and families of people with dementia about the management of dementia symptoms and legal issues.
- Community memory screening initiatives provide free, brief cognitive screenings for older individuals aged 65 years and above. These screenings take place on the islands of Syros, Andros, and Tinos in the Aegean Sea, where the INTRINSIC services are available.

### Ethical considerations

The diagnostic and treatment protocols of both memory clinics adhere to the principles of the sixth revision of the Declaration of Helsinki and received approval from the Bioethics and Research Ethics Committee of the Eginition Hospital of the University of the National and Kapodistrian University of Athens (1,036/31/12/2021, ΑΔΑ 6ΘΞ146Ψ8Ν2-1ΗΙ). Written informed consent was obtained from all beneficiaries or their authorized representatives before being enrolled in INTRINSIC.

## Results

### General practitioner-led memory clinic model

At the GPMemo, 13 people with cognitive complaints were assessed in the first 6 months of its operation. Their demographic and clinical data are presented in Table 1. In terms of comorbidities, nine beneficiaries had three comorbidities, while two individuals had two. The review of the medicines of beneficiaries unveiled 11 cases of polypharmacy, while four people reported falls and four experienced insomnia. Regarding substance use, four regularly consumed alcohol, and three smoked. Hearing loss was reported by 11 individuals. Prior to referral to the memory clinic, only two beneficiaries had received the diagnosis of a mental or neurocognitive disorder, and five had undergone brain imaging. Individuals without brain imaging were referred for a brain CT scan. Performance was abnormal on at least one cognitive screening test in 10 users of the service, while frontal lobe dysfunction was detected in seven beneficiaries (Table 2). Depressive and anxiety symptoms were detected in six and three beneficiaries, respectively.

**Table 1:** Demographic characteristics and somatic disorders of the beneficiaries of the two memory clinics operating in primary healthcare settings in Greece.

	General practitioner-led memory clinic	Registered nurse-led memory clinic
Demographic characteristics		
N	13	27
Age, years*	78 [65–87]	72 [66–82]
Sex (female) (N)	6	18
Education, categories (N)		
Elementary	9	7
Secondary	3	7
3-year higher education	1	11
4 or more years	0	2
Marital status (married)	11	18
Occupational status (retired)	11	22
Living with others	11	19
Somatic diseases (N)		
Hypertension	11	19
Dyslipidemia	10	19
Heart disease	4	9
Diabetes mellitus	3	3
Sum of somatic diseases*	2 [0–3]	3 [1–6]

*Mean [minimum maximum].

**Table 2:** Clinical characteristics of beneficiaries of the two memory clinics operating in primary healthcare settings in Greece.

	General practitioner-led memory clinic	Registered nurse-led memory clinic
Diagnostic tools		
Mini-Cog*	*N* = 13; 3 [0–5]	*N* = 27; 4 [2–5]
Montreal Cognitive Assessment (MOCA)*	*N* = 6; 23 [17–30]	*N* = 27; 24 [15–28]
Mini-Mental State Examination (MMSE)*	*N* = 8; 24 [16–27]	
Frontal Assessment Battery (FAB)*	*N* = 6; 15 [9–17]	
2-item Patient Health Questionnaire (PHQ-2)*	*N* = 13; 2 [0–4]	*N* = 27; 1 [0–3]
15-item Geriatric Depression Scale (GDS)*	*N* = 13; 5 [3–9]	*N* = 26; 7 [0–25]
2-item General Anxiety Disorder (GAD-2)*	*N* = 13; 1 [0–4]	*N* = 27; 1 [0–6]
Beck Anxiety Inventory (BAI)*		*N* = 26; 10 [1–37]
Diagnosis (N)		
Dementia	5	1
MCI	3	8
Depression	1	3
Anxiety disorders	2	0
Sleep disorders	2	0
Dysthymia	0	12
Alcohol	0	1
Mixed anxiety and depression disorder	5	15
Treatment (N)		
Antidementia medicines	6	1
Antidepressants	2	4
Sleep disorder treatment	2	2

*Number of participants, Mean score [minimum maximum].

Five individuals were diagnosed with dementia due to Alzheimer’s disease with vascular changes, three with mild cognitive impairment (MCI), and two of whom were very close to the threshold of dementia. Six individuals received pharmacological treatment for dementia with donepezil, except for one beneficiary in whom memantine was initiated due to contraindications for cholinesterase inhibitors. In addition, in two beneficiaries, treatment for sleep disturbances with melatonin and/or quetiapine was initiated. Two individuals were diagnosed with depression and prescribed citalopram, while one person with anxiety was prescribed trazodone. Individualized counseling focused on increasing physical activity and social empowerment was provided for five cases.

### Nurse-led memory clinic model

At the RNMemo, 27 individuals were assessed and treated in the first 6 months of the operation of the clinic. Among the beneficiaries, eight had three comorbidities, while nine had four comorbidities (Table 1). Interestingly, 13 beneficiaries had been diagnosed with a neuropsychiatric disease prior to their assessment at the RNMemo, i.e., two were diagnosed with an anxiety disorder, six with depression, and eight with mild cognitive impairment. Notably, only six individuals had previously undergone brain imaging. In nine individuals, polypharmacy was detected, 11 reported falls, and 11 had sleep problems. One individual regularly consumed alcohol, and four smoked. Hearing loss was reported in 13 cases. The performance of 20 service users on a neuropsychological assessment tool was abnormal (Table 2). In addition, depressive symptoms were detected in 11 beneficiaries, while five participants screened positive for anxiety disorder on GAD-2 and 14 on BAI (Table 2).

The diagnosis of MCI was established in eight individuals and dementia due to Alzheimer’s disease in one person. In 15 beneficiaries, the diagnosis of mixed anxiety and depression disorder was established. Moreover, 12 people were diagnosed with dysthymia, three with depression, and one individual with alcohol misuse. Pharmacological treatment for dementia was initiated in one case; in four individuals, treatment with antidepressants was started, and melatonin was prescribed for two beneficiaries. In addition, the PATH was recommended to 19 service users who consented to the initiation of psychotherapy.

## Discussion

Despite the surge in dementia prevalence, only one in 10 people with dementia residing in low-income countries receive a diagnosis and have access to treatment and post-diagnostic care (World Health Organization, 2022). Even in the Western world, in low-resource settings, access to quality cognitive health and social care services for older individuals is limited or non-existent (Drew, 2018). To address this gap through the lens of equity, the delivery of services for older individuals with dementia in primary healthcare settings has been advocated as a realistic strategy with universal applicability (Mattap et al., 2022). In this study, the diagnostic and treatment protocols of two different memory clinics, operating in primary healthcare settings, are depicted. One is run by a GP at a primary healthcare center in a semi-mountainous area, while the staff of the other does not include medical healthcare professionals. The latter memory clinic is based on a peripheral general hospital *de facto* operating as a primary healthcare center as well. The diagnostic workup at both clinics covers assessments of cognitive function and neuropsychiatric symptoms (e.g., depression and anxiety). At the GPMemo, performance on activities of daily living is also assessed. Brief instruments were chosen for assessing different symptoms related to neurocognitive disorders, taking into account the difficult balance in primary healthcare between available resources and patient load. Additionally, the time feasibility of the diagnostic workup for cognitive complaints is vital for both the clinician and the examinee during the diagnostic evaluation process in such settings (Georgiou et al., 2022). If memory clinics in primary healthcare are adjusted to this difficult balance, they offer a pragmatic model to address inequities in cognitive healthcare for older individuals (Sen et al., 2022). The data that are reported here provide the first evidence of the feasibility of such models.

Even though the presented memory clinics are still in their infancy, and no accurate conclusions can be drawn, trends of differences between their beneficiaries were observed, which should be treated with caution. For instance, there is a trend of higher prevalence of dementia in beneficiaries of the GPMemo compared to the RNMemo, while the frequency of depressive symptoms does not seem to differ between the two clinics. Of note, a large proportion of the beneficiaries of the RNMemo were diagnosed with depressive and/or anxiety symptoms, i.e., dysthymia or mixed anxiety and depression disorders, while such symptoms were less common in the beneficiaries of the GPMemo. The factors shaping the detected trends warrant investigation in cohorts of adequate sample size. It may be hypothesized that the professional identities of the memory clinic staff (e.g., less reluctance of older individuals residing in low-resource settings to report anxiety and/or depressive symptoms to non-medical healthcare professionals), the characteristics of the local communities (for instance, the RNMemo is located in a more urbanized environment where awareness activities in the community about dementia take place at least once per year, while Erymanthia is a purely rural area, where communities are more dispersed), and the numbers of private neurological and/or psychiatric practices operating in the areas of the memory clinics may be among the factors that have contributed to the detected trends.

The implementation of these two models of memory clinics in primary healthcare settings relies on primary healthcare professionals, such as GPs and nurses, who lacked previous experience in psychogeriatric care before their involvement in the INTRINSIC services. Interestingly, GPs’ contribution to dementia care is still limited, except for the care of people in advanced stages of dementia (Balsinha et al., 2021), and it is criticized for being ineffective and inconsistent (Wangler and Jansky, 2020). In addition, many medical healthcare professionals in primary care face challenges and difficulties with regard to (early) identification of dementia and consistent dementia symptom management, primarily due to limited competence in providing geriatric mental and cognitive care (Lehmann et al., 2018). In INTRINSIC, primary healthcare medical and non-medical professionals attend a series of eight, 1-h, case-based, clinical practice-oriented online seminars delivered by geriatric psychiatrists, experienced GPs, and neuroradiologists, prior to their active involvement in the services. The seminars and other educational materials are available free on the INTRINSIC platform for all professionals of the INTRINSIC services. This pragmatic educational program in combination with the consistent support provided by geriatric psychiatrists via teleconferencing has strengthened the competence of the primary healthcare professionals running the two memory clinics in caring for the cognitive health of older individuals.

Memory clinics in primary healthcare interconnected with tertiary services through telehealth may have the potential for broad application in low-resource settings across the globe. Telehealth is a crucial component of such memory clinics since it facilitates collaboration and communication between dementia specialists, primary healthcare professionals, and beneficiaries and enables the overcoming of geographical barriers. Furthermore, the use of telehealth in these clinics ensures that comorbidities and frailty do not hinder service implementation (Kim, 2017; Gould and Hantke, 2020; Airola, 2021; Elbaz et al., 2021; Goodarzi et al., 2023).

Memory clinics in primary health may be conceived as “one-stop-shop” approach to the management of risk factors for cognitive decline as well as to the diagnosis and treatment of neurocognitive disorders. They are *de facto* multidiagnostic facilities speeding up not only the process of meeting the needs for timely diagnosis and post-diagnostic care of older individuals with dementia but also of managing risk factors for cognitive decline. The pivotal role of primary healthcare professionals in such clinics enables a “one-stop-shop” approach to the management of diseases such as diabetes mellitus, hypertension, dyslipidemia, depression, alcohol misuse/addiction, or conditions such as physical inactivity and obesity, which are well-established risk factors for cognitive decline and dementia. Of note, such services are not expected to be facilities where the emerging disease-modifying treatments for early Alzheimer’s disease will be available, taking into account the high costs of the new agents, the lack of access to biomarkers at low-resource settings, and the demanding monitoring requirements for treatment with these agents (Adewale et al., 2023; Cummings et al., 2023). Nevertheless, at memory clinics in primary healthcare, individuals potentially eligible for such therapies (treatment candidates) can be identified and referred to the centers where such therapies are offered. The strengthening of the competence of primary healthcare professionals to identify people at early stages of Alzheimer’s disease is a pivotal step of paramount importance for primary healthcare-based memory clinics to serve in this capacity. It is noteworthy that there are several barriers to implementing memory clinic services in primary healthcare. For instance, the engagement of primary healthcare professionals in such services pertains to increased work pressure and may subsequently lead to a decrease in motivation. In addition, the continuity of operation of memory clinic services in primary healthcare may be endangered by changes in the primary healthcare center workforce.

The present brief report has several limitations. First, the small numbers of beneficiaries undermine the drawing of accurate conclusions and the comparison of the two memory clinic models in primary healthcare that are described here. Second, the unique characteristics of insular areas might limit the generalizability of the findings to other low-resource communities (Nicolini and Perrin, 2021). Furthermore, comprehensive data on the outcomes of the operation of the presented memory clinics (e.g., beneficiaries’ satisfaction, economic cost, collaboration between healthcare professionals of the primary healthcare centers, and data on interventions) are still being collected. Therefore, additional data are needed to provide further evidence of the acceptability and feasibility of memory clinic services operating in primary healthcare settings.

## Conclusion

The memory clinics that have been developed within the INTRINSIC services in primary healthcare facilities in low-resource settings in Greece rely on telehealth-underpinned interconnections with tertiary services, as well as on a concise, case-based educational program strengthening the competence of primary healthcare professionals to diagnose and manage cognitive decline. The preliminary findings regarding the GPMemo and the RNMemo that are here reported indicate that these models could be applicable to other low-resource primary healthcare settings as well and improve access to essential cognitive health services for older individuals. They reflect a pilot voyage of discovery and experiences that are clearly valuable for all stakeholders advocating and working on the development of memory services embedded in primary healthcare.

## Data Availability

The original contributions presented in the study are included in the article/supplementary material, further inquiries can be directed to the corresponding authors.

## References

[ref1] AbdulRahman M. al-Tahri F. AlMehairi M. K. Carrick F. R. Aldallal A. M. R. (2022). Digital health Technology for Remote Care in primary care during the COVID-19 pandemic: experience from Dubai. Telemed. J. E-Health: Official J. American Telemed. Association 28, 1100–1108. doi: 10.1089/tmj.2021.0459, PMID: 34981953

[ref2] Adewale B. A. Coker M. M. Ogunniyi A. Kalaria R. N. Akinyemi R. O. (2023). Biomarkers and risk assessment of Alzheimer’s disease in low- and middle-income countries. J. Alzheimer’s disease: JAD 95, 1339–1349. doi: 10.3233/JAD-221030, PMID: 37694361

[ref3] Airola E. (2021). Learning and use of eHealth among older adults living at home in rural and nonrural settings: systematic review. J. Med. Internet Res. 23:e23804. doi: 10.2196/23804, PMID: 34860664 PMC8686468

[ref4] Alexopoulos P. Leroi I. Kinchin I. Canty A. J. Dasgupta J. Furlano J. A.. (2024). Relevance and premises of values-based practice for decision making in brain health. Brain Sci. 14:718. doi: 10.3390/brainsci14070718, PMID: 39061458 PMC11274584

[ref5] Alexopoulos P. Novotni A. Novotni G. Vorvolakos T. Vratsista A. Konsta A.. (2020). Old age mental health services in southern Balkans: features, geospatial distribution, current needs, and future perspectives. Eur. Psychiatry 63:e88. doi: 10.1192/j.eurpsy.2020.85 32921324 PMC7576530

[ref6] Arsenault-Lapierre G. Bui T. X. le Berre M. Bergman H. Vedel I. (2023). Rural and urban differences in quality of dementia care of persons with dementia and caregivers across all domains: a systematic review. BMC Health Serv. Res. 23:102. doi: 10.1186/s12913-023-09100-8, PMID: 36721162 PMC9887943

[ref7] Balsinha C. Iliffe S. Dias S. Freitas A. Grave J. Gonçalves-Pereira M. (2021). What is the present role for general practitioners in dementia care? Experiences of general practitioners, patients and family carers in Portugal. Dementia (London, England) 20, 1988–2006. doi: 10.1177/1471301220977710, PMID: 33342279 PMC8358531

[ref8] Cummings J. Apostolova L. Rabinovici G. D. Atri A. Aisen P. Greenberg S.. (2023). Lecanemab: Appropriate Use Recommendations. J. Prev Alzheimers Dis. 10, 362–377. doi: 10.14283/jpad.2023.30, PMID: 37357276 PMC10313141

[ref9] Dautzenberg G. Lijmer J. Beekman A. (2021). Clinical value of the Montreal cognitive assessment (MoCA) in patients suspected of cognitive impairment in old age psychiatry. Using the MoCA for triaging to a memory clinic. Cogn. Neuropsychiatry 26, 1–17. doi: 10.1080/13546805.2020.1850434, PMID: 33272076

[ref10] Drew L. (2018). An age-old story of dementia. Nature 559, S2–S3. doi: 10.1038/d41586-018-05718-5, PMID: 30046085

[ref11] Elbaz S. Cinalioglu K. Sekhon K. Gruber J. Rigas C. Bodenstein K.. (2021). A systematic review of telemedicine for older adults with dementia during COVID-19: an alternative to in-person health services? Front. Neurol. 12:761965. doi: 10.3389/fneur.2021.761965, PMID: 34970210 PMC8712684

[ref12] Fage B. A. Chan C. C. Gill S. S. Noel-Storr A. H. Herrmann N. Smailagic N.. (2021). Mini-cog for the detection of dementia within a community setting. Cochrane Database Syst. Rev. 2021:CD010860. doi: 10.1002/14651858.CD010860.pub3, PMID: 34259337 PMC8278980

[ref13] Ferrari A. J. Santomauro D. F. Aali A. Abate Y. H. Abbafati C. (2023). Global incidence, prevalence, years lived with disability (YLDs), disability-adjusted life-years (DALYs), and healthy life expectancy (HALE) for 371 diseases and injuries in 204 countries and territories and 811 subnational locations, 1990–2021: a systematic analysis for the global burden of disease study 2021. Lancet 403, 2133–2161. doi: 10.1016/S0140-6736(24)00757-8 PMC11122111 38642570

[ref14] Fountoulakis K. N. Tsolaki M. Iacovides A. Yesavage J. O’Hara R. Kazis A.. (1999). The validation of the short form of the geriatric depression scale (GDS) in Greece. Aging (Milan, Italy) 11, 367–372. doi: 10.1007/BF03339814, PMID: 10738851

[ref15] GBD 2019 Dementia Forecasting Collaborators (2022). Estimation of the global prevalence of dementia in 2019 and forecasted prevalence in 2050: an analysis for the global burden of disease study 2019. Lancet Public Health 7, e105–e125. doi: 10.1016/S2468-2667(21)00249-8, PMID: 34998485 PMC8810394

[ref16] Georgiou E. E.-Z. Prapiadou S. Thomopoulos V. Skondra M. Charalampopoulou M. Pachi A.. (2022). Naming ability assessment in neurocognitive disorders: a clinician’s perspective. BMC Psychiatry 22:837. doi: 10.1186/s12888-022-04486-x, PMID: 36585667 PMC9801565

[ref17] Georgiou E.-Z. Skondra M. Charalampopoulou M. Felemegkas P. Pachi A. Stafylidou G.. (2023). Validation of the test for finding word retrieval deficits (WoFi) in detecting Alzheimer’s disease in a naturalistic clinical setting. Eur. J. Ageing 20:29. doi: 10.1007/s10433-023-00772-z, PMID: 37389678 PMC10313575

[ref18] Goodarzi Z. Holroyd-Leduc J. Seitz D. Ismail Z. Kirkham J. Wu P.. (2023). Efficacy of virtual interventions for reducing symptoms of depression in community-dwelling older adults: a systematic review. Int. Psychogeriatr. 35, 131–141. doi: 10.1017/S1041610222000412, PMID: 35603891

[ref19] Gould C. E. Hantke N. C. (2020). Promoting technology and virtual visits to improve older adult mental health in the face of COVID-19. Am. J. Geriatr. Psychiatry 28, 889–890. doi: 10.1016/j.jagp.2020.05.011, PMID: 32425468 PMC7227501

[ref20] Kalaria R. Maestre G. Mahinrad S. Acosta D. M. Akinyemi R. O. (2024). The 2022 symposium on dementia and brain aging in low- and middle-income countries: highlights on research, diagnosis, care, and impact. Alzheimers Dement. 20, 4290–4314. doi: 10.1002/alz.13836, PMID: 38696263 PMC11180946

[ref21] Kanellopoulos D. Rosenberg P. Ravdin L. D. Maldonado D. Jamil N. Quinn C.. (2020). Depression, cognitive, and functional outcomes of problem adaptation therapy (PATH) in older adults with major depression and mild cognitive deficits. Int. Psychogeriatr. 32, 485–493. doi: 10.1017/S1041610219001716, PMID: 31910916 PMC7165030

[ref22] Kim H. (2017). The effect of telemedicine on cognitive decline in patients with dementia. J. Telemed. Telecare 23, 149–154. doi: 10.1177/1357633X15615049 26541171

[ref23] Kourtesis P. Margioti E. Demenega C. Christidi F. Abrahams S. (2020). A comparison of the Greek ACE-III, M-ACE, ACE-R, MMSE, and ECAS in the assessment and identification of Alzheimer’s disease. J. Int. Neuropsycholog. Society: JINS 26, 825–834. doi: 10.1017/S1355617720000314, PMID: 32312343

[ref24] Krause K. E. Kokorelias K. M. Sinha S. K. (2022). A systematic review and qualitative analysis of geriatric models of care for rural and remote populations. Rural Remote Health 22:7486. doi: 10.22605/RRH7486, PMID: 35975281

[ref25] Lahana E. Pappa E. Niakas D. (2011). Do place of residence and ethnicity affect health services utilization? Evidence from Greece. Int. J. Equity Health 10:16. doi: 10.1186/1475-9276-10-16, PMID: 21521512 PMC3107789

[ref26] Lehmann J. Michalowsky B. Kaczynski A. Thyrian J. R. Schenk N. S. Esser A.. (2018). The impact of hospitalization on readmission, institutionalization, and mortality of people with dementia: a systematic review and Meta-analysis. J. Alzheimers Dis. 64, 735–749. doi: 10.3233/jad-171128, PMID: 29966191

[ref28] Livingston G. Huntley J. Sommerlad A. Ames D. Ballard C. Banerjee S.. (2020). Dementia prevention, intervention, and care: 2020 report of the lancet commission. Lancet (London, England) 396, 413–446. doi: 10.1016/S0140-6736(20)30367-6, PMID: 32738937 PMC7392084

[ref29] Mattap S. M. Mohan D. McGrattan A. M. Allotey P. Stephan B. C. M. Reidpath D. D.. (2022). The economic burden of dementia in low- and middle-income countries (LMICs): a systematic review. BMJ Glob. Health 7:e007409. doi: 10.1136/bmjgh-2021-007409, PMID: 35379735 PMC8981345

[ref30] Nicolini M. Perrin T. (2021). Geographical connections: law, islands, and remoteness. Liverpool Law Rev. 42, 1–14. doi: 10.1007/s10991-020-09259-8

[ref31] Panagiotopoulos G. Kaliampakos D. (2019). Accessibility and spatial inequalities in Greece. Appl. Spat. Anal. Policy 12, 567–586. doi: 10.1007/s12061-018-9256-8

[ref32] Politis A. M. Mayer L. S. Passa M. Maillis A. Lyketsos C. G. (2004). Validity and reliability of the newly translated Hellenic neuropsychiatric inventory (H-NPI) applied to Greek outpatients with Alzheimer’s disease: a study of disturbing behaviors among referrals to a memory clinic. Int. J. Geriatr. Psychiatry 19, 203–208. doi: 10.1002/gps.1045, PMID: 15027034

[ref33] Politis A. Vorvolakos T. Kontogianni E. Alexaki M. Georgiou E. Z. Aggeletaki E.. (2023). Old-age mental telehealth services at primary healthcare centers in low- resource areas in Greece: design, iterative development and single-site pilot study findings. BMC Health Serv. Res. 23:626. doi: 10.1186/s12913-023-09583-5, PMID: 37312113 PMC10262121

[ref34] Poptsi E. Moraitou D. Eleftheriou M. Kounti-Zafeiropoulou F. Papasozomenou C. Agogiatou C.. (2019). Normative data for the Montreal cognitive assessment in Greek older adults with subjective cognitive decline, mild cognitive impairment and dementia. J. Geriatr. Psychiatry Neurol. 32, 265–274. doi: 10.1177/0891988719853046, PMID: 31159629

[ref35] Prince M. Guerchet M. Prina M. (2015) ‘The epidemiology and impact of dementia - current state and future trends. WHO Thematic Briefing’. Available at: https://hal.science/hal-03517019 (Accessed on 21 December 2023).

[ref36] Sachdev P. S. Blacker D. Blazer D. G. Ganguli M. Jeste D. V. Paulsen J. S.. (2014). Classifying neurocognitive disorders: the DSM-5 approach. Nat. Rev. Neurol. 10, 634–642. doi: 10.1038/nrneurol.2014.181, PMID: 25266297

[ref37] Sapra A. Bhandari P. Sharma S. Chanpura T. Lopp L. (2020). Using generalized anxiety Disorder-2 (GAD-2) and GAD-7 in a primary care setting. Cureus 12:e8224. doi: 10.7759/cureus.8224, PMID: 32582485 PMC7306644

[ref38] Sen K. Prybutok G. Prybutok V. (2022). The use of digital technology for social wellbeing reduces social isolation in older adults: a systematic review. SSM - Population Health 17:101020. doi: 10.1016/j.ssmph.2021.101020, PMID: 35024424 PMC8733322

[ref39] Shin J. H. (2022). Dementia epidemiology fact sheet 2022. Ann. Rehabil. Med. 46, 53–59. doi: 10.5535/arm.22027, PMID: 35508924 PMC9081392

[ref40] Slachevsky A. Villalpando J. M. Sarazin M. Hahn-Barma V. Pillon B. Dubois B. (2004). Frontal assessment battery and differential diagnosis of frontotemporal dementia and Alzheimer disease. Arch. Neurol. 61, 1104–1107. doi: 10.1001/archneur.61.7.1104 15262742

[ref41] Sorinmade O. A. Kossoff L. Peisah C. (2020). COVID-19 and telehealth in older adult psychiatry-opportunities for now and the future. Int. J. Geriatr. Psychiatry 35, 1427–1430. doi: 10.1002/gps.5383, PMID: 32729632

[ref42] Suhr J. A. Angers K. (2019). “Neuropsychological testing and assessment” in The Cambridge handbook of clinical assessment and diagnosis. eds. Suhr J. A. Sellbom M. (Cambridge: Cambridge University Press (Cambridge Handbooks in Psychology)), 191–207.

[ref43] Thompson C. Halcomb E. Masso M. (2023). The contribution of primary care practitioners to interventions reducing loneliness and social isolation in older people-an integrative review. Scand. J. Caring Sci. 37, 611–627. doi: 10.1111/scs.13151, PMID: 36732897

[ref44] UNDP (2017). Ageing, older persons and the 2030 agenda for sustainable development. Available at: https://www.undp.org/publications/ageing-older-persons-and-2030-agenda-sustainable-development (Accessed on 21 December 2023).

[ref46] Wahlund L.-O. (2016). Imaging biomarkers of dementia: recommended visual rating scales with teaching cases. Insights Imag. 8, 79–90. doi: 10.1007/s13244-016-0521-6, PMID: 28004274 PMC5265189

[ref47] Wangler J. Jansky M. (2020). Dementia diagnostics in general practitioner care: do general practitioners have reservations? The findings of a qualitative study in Germany. Wiener Medizinische Wochenschrift (1946) 170, 230–237. doi: 10.1007/s10354-019-00722-4, PMID: 31811446 PMC7272384

[ref48] World Health Organization (2017). Global strategy and action plan on ageing and health. Geneva: World Health Organization.

[ref49] World Health Organization (2022). Optimizing brain health across the life course: WHO position paper. Geneva: World Health Organization.

[ref50] World Health Organization and Wonca Working Party on Mental Health (2008). What is primary care mental health? Ment Health Fam Med 5, 9–13. 22477841 PMC2777553

